# Prospective Randomized Evaluation of Decompressive Ipsilateral Craniectomy for Traumatic Acute Epidural Hematoma (PREDICT-AEDH): study protocol for a randomized controlled trial

**DOI:** 10.1186/s13063-021-05359-6

**Published:** 2021-06-29

**Authors:** Chun Yang, Xianjian Huang, Junfeng Feng, Li Xie, Jiyuan Hui, Weiping Li, Jiyao Jiang

**Affiliations:** 1 Brain Injury Center, Department of Neurosurgery, Renji Hospital, School of Medicine, Shanghai Jiao Tong University, 160 Pujian Road, Shanghai, People’s Republic of China; 2 Shanghai Institute of Head Trauma, Shanghai, People’s Republic of China; 3 Department of Neurosurgery, The First Affiliated Hospital of Shenzhen University, 3002 Sungang West Road, Shenzhen, Guangdong People’s Republic of China; 4 Clinical Research Institute, Shanghai Jiao Tong University School of Medicine, 227 Chongqing South Road, Shanghai, People’s Republic of China

**Keywords:** Acute epidural hematoma (AEDH), Herniation, Surgery, Decompressive craniectomy (DC), Craniotomy, Randomized controlled trial (RCT)

## Abstract

**Background:**

The expeditious surgical evacuation of acute epidural hematoma (AEDH) is an attainable gold standard and is often expected to have a good clinical outcome for patients with surgical indications. However, controversy exists on the optimal surgical options for AEDH, especially for patients with brain herniation. Neurosurgeons are confronted with the decision to evacuate the hematoma with decompressive craniectomy (DC) or craniotomy.

**Methods/design:**

Patients of both sexes, age between 18 and 65 years, who presented to the emergency room with a clinical and radiological diagnosis of AEDH with herniation, were assessed against the inclusion and exclusion criteria to be enrolled in the study. Clinical and radiological information, including diagnosis of AEDH, treatment procedures, and follow-up data at 1, 3, and 6 months after injury, was collected from 120 eligible patients in 51 centers. The patients were randomized into groups of DC versus craniotomy in a 1:1 ratio. The primary outcome was the Glasgow Outcome Score-Extended (GOSE) at 6 months post-injury. Secondary outcomes included incidence of postoperative cerebral infarction, incidence of additional craniocerebral surgery, and other evaluation indicators within 6 months post-injury.

**Discussion:**

This study is expected to support neurosurgeons in their decision to evacuate the epidural hematoma with or without a DC, especially in patients with brain herniation, and provide additional evidence to improve the knowledge in clinical practice.

**Trial registration:**

ClinicalTrials.govNCT 04261673. Registered on 04 February 2020

## Background

Traumatic brain injury (TBI) represents the most challenging global public health care issues, with more than 50 million people suffering from TBI each year worldwide [1, 2]. Acute epidural hematoma (AEDH), a common and severe type of TBI, occurring approximately in 2 to 4% of all TBI patients [3–5], accounts for a substantial proportion of fatal head injuries, with mortality ranging from 1.2 to 33%. AEDH befalls more frequently in young people, with a mean age between 20 and 40 years. Elderly people rarely suffer from AEDH but have a significantly higher mortality. Vehicle-related accidents are the main cause of AEDH, accounting for 53% of all cases (range, 30–86%). Other causes of AEDH, including falls, assaults, and sports injuries, have been reported in several studies [3–5].

Epidural hematomas are caused by bleeding between the outer layers covering the brain and the inner table of the skull. The rapid rise of intracranial pressure (ICP), caused by the mass effect, will result in midline shift and direct or indirect compression of the brainstem. This condition represents a potentially life-threatening emergency and requires prompt diagnosis and neurosurgical intervention. The large availability of computed tomography (CT) scanners for internal examinations facilitates the fast diagnosis of AEDH and allows the monitoring of the disease progression. Thus, the expeditious surgical evacuation of AEDH is an attainable gold standard and is often expected to have a good clinical outcome. Indeed, the treatable nature of epidural hematomas (EDH) has led some surgeons to suggest a “toward zero mortality” as an achievable target for this condition.

Ordinary surgical strategy principally aims to quickly reduce ICP and promote the repositioning of the brain herniation, if concurrent [3]. The Brain Trauma Foundation (BTF) has produced an informative guideline on the surgical indications for AEDH, but no specific surgical options. Although craniotomy for AEDH is conventionally employed in practice, some patients suffered from clinical deterioration by secondary injuries, including fatal cerebral infarction (CI), especially in cases combined with preoperative herniation [3]. In such cases, an initial hematoma evacuation with additional decompressive craniectomy (DC) might effectively prevent and/or alleviate postoperative CI. However, the relatively low incidence of postoperative CI has led few surgeons to opt for the removal of the bone flap in patients with AEDH. In addition, the lack of high-quality evidence regarding the use of decompressive craniectomy in AEDH has determined a great variability in terms of practices between hospitals, countries, and even among surgeons within the same hospital. Other critical issues, such as the proper identification of subgroups that may not benefit from DC, are in need of further exploration. To date, there are no clinical trials that have investigated the validity of decompressive craniectomy in AEDH patients with herniation.

In this research, called Prospective Randomized Evaluation of Decompressive Ipsilateral Craniectomy for Traumatic Acute Epidural Hematoma (PREDICT-AEDH), we present a study protocol for a randomized controlled trial. Our aim is to compare the outcome and cost-effectiveness of DC versus craniotomy in the treatment of TBI patients with cerebral herniation undergoing acute epidural hematoma evacuation.

## Methods and design

### Study design

**Fig. 1:**
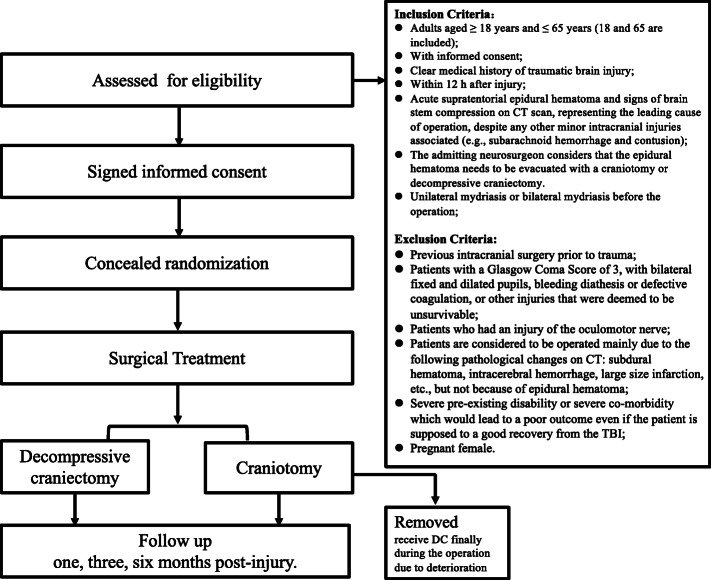
Study flow chart

This is a study protocol for a randomized, multicenter, clinical controlled, pragmatic trial involving 51 neurosurgical centers. Two intervention arms are designed, where patients are allocated in both the craniotomy and DC groups. The patients’ enrollment is expected to be conducted from September 2020 to October 2023 within all the centers involved. All patients are expected to be observed for a total period of 6 months, and the last 6 months’ follow-up assessment will end in May 2024. Clinical information of enrolled participants, including diagnosis of AEDH, clinical radiological information, treatment procedures, and follow-up data, will be collected in detail. A concise flow chart, displaying the overall study design, is shown in Fig. 1. The detailed schedule regarding enrollment, interventions, assessments, and visits for participants is shown in Table 1.

**Table 1:** Time schedule of enrollment, interventions, assessments, and follow-up for participants

Time points	Enrollment	Operation and treatment	Follow-up			Adverse effects and other operations
	Day 0	Day 0 to discharge	1 month post-injury	3 months post-injury	6 months post-injury	Within 6 months post-injury
**Informed consent**	**X**					
**Eligibility**	**X**					
**Information of enrollment**	**X**					
**Patient information**	**X**					
**Medical history**	**X**					
**Randomization**	**X**					
**Surgery notes**		**X**				**X**
**Physical and neurological examination**	**X**	**X**	**X**	**X**	**X**	
**Imaging**	**X**	**X**	**X**	**X**	**X**	**X**
**ICP management**		**X**				
**GOSE**		**X**	**X**	**X**	**X**	
**LOS**		**X**	**X**	**X**	**X**	
**Treatment cost**		**X**	**X**	**X**	**X**	
**MMSE**			**X**	**X**	**X**	
**EQ-5D-5L**			**X**	**X**	**X**	

### Participant selection

Patients presenting to the emergency room, with a clinical and radiological diagnosis of AEDH, are eligible for inclusion. The eligible criteria of each patient will be determined by the senior neurosurgeon from each participating center and an external radiologist not involved in the allocation process. This study specifically focuses on patients with unilateral supratentorial epidural hematoma. Herniation here mainly refers to uncal herniation, a common subtype of transtentorial herniation, frequently caused by AEDH located in the temporoparietal and temporal regions. The main criteria to identify uncal herniation are pupillary enlargement and signs of brain stem compression on CT scans.

#### Inclusion criteria

- ➢ Adults aged ≥ 18 years and ≤ 65 years (18 and 65 are included).
- ➢ With informed consent.
- ➢ Clear medical history of traumatic brain injury.
- ➢ Within 12 h after injury.
- ➢ Acute supratentorial epidural hematoma and signs of brain stem compression on CT scan, representing the leading cause of operation, despite any other minor intracranial injuries associated (e.g., subarachnoid hemorrhage and contusion).
- ➢ The admitting neurosurgeon considers that the epidural hematoma needs to be evacuated with a craniotomy or decompressive craniectomy.
- ➢ Unilateral mydriasis or bilateral mydriasis before the operation.

#### Exclusion criteria

- ➢ Previous intracranial surgery before trauma.
- ➢ Patients with a Glasgow Coma Score of 3, with bilateral fixed and dilated pupils, bleeding diathesis or defective coagulation, or other injuries that were deemed to be unsurvivable.
- ➢ Patients who had an injury to the oculomotor nerve.
- ➢ Patients are considered to be operated mainly due to the following pathological changes on CT: subdural hematoma, intracerebral hemorrhage, large size infarction, etc., but not because of epidural hematoma.
- ➢ Severe pre-existing disability or severe co-morbidity leading to a poor outcome even if the patient is supposed to have a good recovery from the TBI.
- ➢ Pregnant female.

### Sample size calculation, randomization, and blinding

Our data and results from a previous retrospective study are showing that almost all the patients who received a synchronous DC (93%) had a GOSE of 6.63 ± 1.27, with a favorable outcome (defined by the GOSE categories: lower moderate disability, upper moderate disability, lower good recovery, and upper good recovery). Patients who received craniotomy had a GOSE of 5.78 ± 1.58, with a favorable outcome in 72% of the patients [6]. We calculated the sample required in this clinical trial with a significance level of 5% (two-sided) and a power of 80% to demonstrate the difference in the rate of favorable outcome, with an assumption that 10% of the patients would be lost during follow-up. Ultimately, considering the quality of the study, the sample size is enlarged to 120.

All participating centers must have a neurosurgical on-call team and an emergency department responsible for the admission of head-injured patients to identify eligible patients. After recording the patient’s clinical information, including Glasgow Coma Scale (GCS), pupillary reactivity, and CT findings, an electronic data collection (EDC) database will generate the subject identification number that will be allocated to the eligible patients. A secure central web-based randomization management information system will be used for the randomization of eligible patients. The system will provide an immediate allocation along with the patient randomization record number for the trial, and individual randomization record numbers can be checked at any time. Blocked randomization will be used, with a block size of 4 and an allocation ratio of 1:1. The 24-h central web-based randomization management information system will be backed by one delegated person (available 24 h per day) who will assure the cover-up of the record allocation. The delegated person responsible for randomization will not be involved in the treatment procedure. A confirmatory email will be sent to the investigator of each center involved to verify the allocation.

A patient who is randomly allocated to the craniotomy group, but finally receives a decompressive craniectomy as a lifesaving action during the initial surgery, will be rejected from this randomized controlled trial. This potential selection bias is beyond the control of the investigators, and according to the current guidelines, no specific surgical options are provided for the enrolled participants. Nevertheless, patients’ detailed data will be recorded, providing the observational cohort information necessary to further analyze the possible factors and potential bias.

Due to the nature of the intervention, and the risk that DC surgical procedure will result in skull defect, patients and treating doctors cannot be blinded to allocation. However, the evaluators involved in this study and the investigators responsible for the outcomes’ analysis are blinded to the groups.

### Treatment interventions

The randomization process allocates eligible patients to one of the two surgical treatment groups. Surgical options, according to the guidelines for the surgical management of AEDH [7], consist of the evacuation of the hematoma with a craniotomy, or with additional DC, leaving a large bone flap [3]. However, the bone flap must be replaced and secured with a fixation system (plates and screws) in patients who received a craniotomy. It is essential that all treatments for surgical management of AEDH are consistent within the two groups, including preoperative preparation and anesthesia. The only difference between the two arms will be represented by the removal or replacement of the bone flap. To provide well-established surgical techniques and a standardized surgical treatment is the essential aim of this study. The operation will be performed by a qualified senior neurosurgeon within each of the participating centers.

The post-operation management process and the medical decisions concerning the patients will not be affected by their recruitment into the PREDICT study. All patients will receive postoperative care management according to the guidelines and intensive care protocols, including radiographic or biochemical examination. Normally, a cranioplasty is recommended to reconstruct the skull in patients who underwent DC. The treatment in case of complications will differ considerably between patients, according to their clinical manifestations (epilepsy or cerebral infarction, or late complications as hydrocephalus).

### Aims and outcomes

The trial aims to evaluate craniotomy and DC on AEDH patients in terms of efficacy, economic benefits, and complications. The primary endpoint is the Glasgow Outcome Score-Extended (GOSE) at 6 months post-injury, the most commonly used prognostic evaluation scale in TBI, which allows the assessment of the long-term functional outcomes, including overall mortality and morbidity rates [8, 9].

Additionally, the following secondary endpoints are intended to be investigated as supplementary functional and cognitive measures:

1. The incidence of postoperative cerebral infarction within 6 months post-injury, to be primarily diagnosed by independent radiologists by CT or MRI examination
2. The incidence of additional craniocerebral surgery, as a result of clinical deterioration after initial surgical treatment for AEDH, within 6 months post-injury
3. The incidence of serious adverse events (SAE) within 6 months post-injury, and SAE is defined as an untoward occurrence that:
  - Results in death
  - Is life-threatening
  - Requires hospitalization or prolongation of existing hospitalization
  - Results in persistent or significant disability or incapacity
  - Is otherwise considered medically significant by the investigator
4. The length of patient’s stay in ICU and hospital after initial surgery within 6 months post-injury
5. Detailed economic evaluation, defined as the total medical expenses related to the treatment for AEDH, including the costs of operations, hospitalization, and rehabilitation within 6 months post-injury
6. Quality of life at 6 months post-injury with the score of 5-level EuroQol Five Dimensions Questionnaire (EQ-5D-5L). The EQ-5D is a generic instrument to describe and evaluate the health condition on the basis of five dimensions: mobility, self-care, usual activities, pain/discomfort, and anxiety/depression
7. MMSE (Mini-Mental State Examination) scores at 6 months post-injury

### Data collection

Once patients are enrolled in the study, data related to demographic and clinical information, including hospital admission, treatment process, and follow-up, will be collected by the local investigators and recorded on the case report form (CRF). Detailed data consisting of neurological condition, timing of injury (also the ER, initial CT, and surgical operation), radiographic abnormalities, operation data, and postoperative care management (including ICP management and pharmacological data) will also be recorded. Postoperative CT scan will be performed routinely on the 1st, 3rd, and 7th day post-initial surgery. Cerebral infarction will be initially detected by postoperative CT image depicting a low-dense area. The blood supply of involved lobes will be further detected by transcranial Doppler sonography (TCD) and/or MR. Follow-up visits in the outpatient department are scheduled at 1, 3, and 6 months after injury; data will be collected and recorded. The information recorded on CRFs will be entered into an electronic data collection (EDC) database by a designated person within each participating center. A subject must be withdrawn from the trial if one of the following applies:

1. Each patient or his/her legally authorized representative has the right to require withdrawal from the study cohort for any reasons after enrollment.
2. The investigator will withdraw a patient who is randomly allocated to a craniotomy and that eventually requires a DC as lifesaving action during the initial surgery.
3. In addition, the investigator could withdraw the patients from their allocated treatment group if a clinical reason for not performing the surgical intervention is identified after randomization.
4. Lost to follow-up.

The primary reason for all discontinuations and withdrawals will be documented.

### Implementation and data management

A Trial Management Committee (TMC), including chief investigators from each site, clinical research associates (CRA), the ethics committee and institutional review board, and treatment representatives, is established to ensure a proper implementation throughout the trial process. The chief investigators will be responsible for ensuring adherence to the study protocol, compliance with the consent process, follow-up, and accurate data collection. The trial coordinating center, authorized by TMC, will report all the enrollment records and schemes every month and will monitor the recruitment phase and assess the trial progresses in collaboration with the clinical investigators. A research summary conference will be held every 6 months to evaluate progresses and discuss possible solutions to the problems that may arise.

All data will be collected and recorded using CRFs and the EDC; a data management committee (DMC, located at Clinical Research Institute, Shanghai Jiaotong University School of Medicine) will be established to develop and maintain the EDC and supervise the data quality. Monitoring and auditing will be performed against the original documentation on a monthly basis by the CRA of DMC. Regular visits will be scheduled in each participating center to ensure the compliance with the research program requirements. In case of anomalies, the CRA will promptly submit the information to the investigators in order to find a solution.

All investigators participating in this study will comply with the requirements of the Data Protection Act 1998 concerning the collection, storage, processing, and disclosure of personal information. Access to collated participant data will be restricted to approved individuals involved in the treatment process and representatives from regulatory authorities. Computers used to input data will set up user names and passwords with limited access measures. Published results will not contain any personal data that could allow the participants’ identification. All study documents will be kept for a minimum of 5 years, and documents will not be destroyed without permission from the leading research center when the minimum retention period has elapsed.

### Data analysis

Patient characteristics and variations in both treatments and outcomes will be defined using descriptive statistics. Continuous variables will be described as mean and standard deviation (SD) or as median and interquartile range (IQR). To assess the differences between cohorts, appropriate tests will be performed according to the distribution and scale of measurement. Student’s t-tests or Mann-Whitney U tests will be used for continuous variables, and *X*^*2*^ tests or Fisher’s exact test will be used for categorical variables. Exploratory analyses on AEDH will be performed, aiming at understanding the complexity of the disease and to discover new possible associations. In addition to standard descriptive statistics, multivariable regression models or other analyses may be used if appropriate. *P* < 0.05 will be considered to be statistically significant. Factors such as age, the severity of injury, and the timing of injury will be included as covariates in the primary analysis, with the center considered as a random effect.

More specifically, the outcome measures will be assessed using the GOSE, an ordinal scale. The GOSE categories of death, vegetative state, lower severe disability, and upper severe disability will be considered as unfavorable outcomes. Favorable outcomes will be defined by following the GOSE categories: lower moderate disability, upper moderate disability, lower good recovery, and upper good recovery. However, GOSE conventional analysis usually dichotomizes the ordinal scale into a binary scale, and this may discard a lot of relevant information, reducing both the outcomes’ clinical relevance and the statistical efficiency of the analysis. Ordinal logistic regression analysis will be used to quantify the prognostic effects across the full range of the GOSE. And the final effect will be a pooled estimate of the common odds ratio. Additionally, a secondary analysis will be reported for the two pre-specified binary outcomes: dead versus alive and unfavorable versus favorable outcomes.

So far, there is no reliable data able to clarify the potential selection bias caused by the number of patients who, after initial allocation to a craniotomy, eventually require a DC. This study aims to be a pragmatic trial to improve and specify the surgical options for AEDH. Data of patients who, after initially undergoing craniotomy, eventually require a DC will be recorded, as the incidence of additional craniocerebral surgery after the initial surgical treatment (secondary endpoint). All these information will help in evaluating the potential selection bias.

### Ethics and dissemination

The study protocol has been approved by the ethics committee and institutional review board of Renji Hospital, School of Medicine, Shanghai Jiao Tong University (KY2020-038), representing the center that supports this research. Other participating centers will be separately evaluated in their participation from each ethics committee or institutional review board. During the research process, the study investigators will strictly follow the Declaration of Helsinki and Human Biomedical Research Ethical Issues. Any protocol amendments will be first submitted to the review board for approval. The management process and medical treatment of the enrolled participants will not be affected by their recruitment into the PREDICT study. In case of death or occurrence of serious adverse events, a detailed report will be sent to the ethics committee and institutional review board of the leading research center.

All enrolled participants will be asked to provide a signed informed consent to produce documentary evidence on the information received on the clinical trial, the study interventions, participants’ rights, and the voluntary nature of their participation. Predictably, most eligible participants will be incapacitated to give consent for trial entry themselves, due to the brain injury. If the next of kin are available, they will be asked to give proxy consent for the patient’s admission. A brief discussion explaining the patient’s participation in the study will take place face to face or by telephone. If the next of kin are unavailable or cannot be reached before the operation, the principal investigator will take responsibility for including the patient into the trial, provided that the following conditions are met:

1. The patient is eligible for trial entry.
2. The patient is in a life-threatening situation and an urgent operation is required without delay.
3. Operation is not detachable from decompressive craniectomy or craniotomy during the treatment of a patient undergoing evacuation of an acute epidural hematoma.
4. Both decompressive craniectomy and craniotomy are well established in the treatment center and routinely performed by the investigator.
5. Investigator documents in the declaration form are approved by the ethics committee and institutional review board of the treatment center.

Best efforts will be made to contact the next of kin during the treatment and seek their approval to the patient’s inclusion within the trial. Views of withdrawal to enter or continue the trial from next of kin will be respected, and no further data will be collected from the patient.

If participants regain their decisional capacity during the procedure, they will be given information about the trial and their consent will be sought to continue in the trial. Meanwhile, participants or next of kin are also informed that they could withdraw consent and quit at any moment during trial, without affecting their treatment process, only by informing the investigator on their willingness to keep their collected data for future analysis or delete them.

No patients or the public were directly involved in the design or conduct of the study. The patients and their caregivers will be informed about the whole duration of the study (4 years), and any development may occur during the process. The trial results will be disseminated through academic conferences and published in peer-reviewed journals. In case of publication, the investigators will immediately inform the patients and their caregivers by telephone or e-mail.

## Discussion

This study will be the first randomized controlled trial designed to evaluate the benefits of DC on traumatic AEDH patients with brain herniation [10, 11]. Two different treatment methods, decompressive craniectomy and craniotomy, will be compared, assessing the discriminative option of removing or replacing the bone flap. The study outcomes will possibly clarify the best indications in case of decompressive craniectomy and will support the decision-making during preoperative surgical planning in AEDH cases.

There is a considerable controversy regarding the initial neurosurgical management of AEDH [4, 12, 13]. In some cases, neurosurgeons are confronted with the decision to evacuate the hematoma with or without a DC, especially in patients with brain herniation [6, 14]. The BTF recommends that all patients with an EDH volume of more than 30 cm^3^ should undergo surgical evacuation of hematoma, regardless of Glasgow Coma Scale [3]. Although no sufficient data are available to support certain surgical treatment methods, craniotomy provides a more complete evacuation of the hematoma [3]. However, evidence is showing that a set of patients experienced clinical deterioration after the initial hematoma-evacuation craniotomy, as a result of secondary brain injuries with increased ICP [10, 15–18]. Additionally, the incidence of post-traumatic cerebral infarction secondary to AEDH is reported to be 18.2% and even higher among patients with high-risk factors, e.g., transtemporal location, preoperative shock for longer than 30 min, bilateral mydriasis, preoperative brain herniation, and so on [3, 6, 19]. Nevertheless, decompressive craniectomy has displayed a potential in controlling raised ICP and is recommended as soon as possible in case of serious post-traumatic cerebral infarction secondary to AEDH [6, 10, 15, 17–19]. DC seems to represent the best surgical option in some cases of AEDH. However, previous evidence from clinical practice indicates that bone flap removal is not always essential in patients with EDH [5, 20]. DC performed inappropriately with initial hematoma-evacuation might lead to unavoidable complications, such as abnormal hemodynamics, subsequent cerebral necrosis, and infarction, as well as a need for cranioplasty [15, 21]. Inappropriate management with undefined surgical indications can lead to unnecessary death and disability, which may defeat the target of “toward zero mortality” while managing AEDH. To date, no consensus has been reached around the opportunity to proceed with decompressive craniectomy in the management of AEDH with brain herniation. And also, regarding the type of surgical techniques during decompressive craniectomy, it is in need of further investigation, by mean of randomized controlled trials [10].

China is a large country with a wide population distribution. Primary hospitals are the main battleground of TBI, and where the management of AEDH, with characteristics of relatively high incidence and likely rapid deterioration, needs to be improved. Fast diagnosis and effective management for AEDH are the most important tools for medical workers in primary hospitals. Therefore, in current surgical practice patterns, there is a clinical rationale to investigate DC while dealing with AEDH and enhance the general lack of evidence. This study is expected to help neurosurgeons in their decisions to evacuate the epidural hematoma with or without a DC, especially with cases of brain herniation, and to provide a strong level of evidence for the surgical management of AEDH.

## Trial status

Recruitment started in September 2020 and is planned to end in October 2023, with 120 patients randomized. The follow-up period will end in May 2024. The current protocol version is 1.0, dated 8 February 2021.

## Data Availability

Not applicable.

## Abbreviations

AEDH: Acute epidural hematoma
TBI: Traumatic brain injury
GOSE: Glasgow Outcome Score-Extended
DC: Decompressive craniectomy
RCT: Randomized controlled trial
EDH: Epidural hematoma
ICP: Intracranial pressure
BTF: Brain Trauma Foundation
CI: Cerebral infarction
SAE: Serious adverse events
EQ-5D: EuroQol Five Dimensions Questionnaire
MMSE: Mini-Mental State Examination
ER: Emergency room
TCD: Transcranial Doppler sonography
EDC: Electronic data collection
DMC: Data management committee
SD: Standard deviation
IQR: Interquartile range
